# Paternally derived translocation t(8;18)(q22.1;q22)pat associated in a patient with developmental delay: Case report and review

**DOI:** 10.4103/1817-1745.66686

**Published:** 2010

**Authors:** Lakshmi Rao, Murthy Kanakavalli, Venkata Padmalatha, Pratibha Nallari, Lalji Singh

**Affiliations:** Centre for Cellular and Molecular Biology, Uppal Road, Hyderabad, India; 1 Department of Genetics, Osmania University, Hyderabad, India

**Keywords:** Balanced reciprocal translocation, chromosomes 8 and 18, delayed milestones, Giemsa–Trypsin–Giemsa banding and FISH, translocation carrier

## Abstract

The common cause of mental impairment and the wide range of physical abnormalities is balanced chromosome rearrangement. As such, it is difficult to interpret, posing as a diagnostic challenge in human development. We present a unique familial case report with the paternally inherited autosomal-balanced reciprocal translocation involving chromosomal regions 8q and 18q. The etiology of the translocation, *i.e.* 46,XX,t(8;18)(q22.1;q22) was detected by conventional high-resolution Giemsa–Trypsin–Giemsa-banding and fluorescence *in situ* hybridization techniques. The father was found to be the carrier of the chromosome defect and also the same was observed in the first female child referred with a history of delayed milestone development. However, the second female child showed normal 46, XX karyotype. This is the first report of reciprocal translocation involving 8q and 18q associated with the delayed milestone development. The reason likely may be due to the rearrangement of genetic material at these breakpoints having a crucial relationship and thus manifesting developmental delay in the progeny. Accordingly, this paper also shows genetic counseling discussion for the cause.

## Introduction

In humans, the incidence of balanced chromosome translocations is approximately one in 500.[1] In cytogenetic evaluation, balanced chromosome translocations are defined as those rearrangements where no loss or gain of genetic material is observed. Most of the balanced chromosome rearrangements (BCRs) are not considered to be associated with the clinical (phenotype) abnormalities. But, they are of concern, as the carriers of BCRs offer a greater risk to their descendents with congenital anomalies or recurrent miscarriages. On the basis of a few evaluation procedures, it is estimated that 6.7% of the carriers of *de novo* BCRs have the risk of phenotypic abnormalities.[2] Most of the hypotheses that have been proposed to explain the association of BCRs with phenotypic abnormalities[3] include monogenic disorder, such as sickle cell anemia caused by modification or disruption of genes, unbalanced rearrangement at the molecular level, mosaic or varied chromosomal complement for unbalanced rearrangement in another tissue, uniparental disomy of one of the chromosomes involved in the rearrangement and position effect variegation phenomenon.

Almost all the carriers of the balanced reciprocal translocations are believed to be normal by phenotype. Moreover, it is known that the modification or inactivation of specific disease genes at chromosomal breakpoints have been very phenomenal in identifying genes that are associated with a variety of disorders, mostly early-onset disorders.[4] In the present paper, we describe paternally inherited autosomal-balanced reciprocal translocation involving chromosomes 8 and 18 in the progeny associated with delayed milestone development.

## Case Report

The proband is a 3-year-old girl, the first female child of a healthy, young, nonconsanguineous couple. Pregnancy and delivery at 38 weeks were normal. Birth weight was 2,800 grams. The proband was referred for cytogenetic evaluation with a history of delayed milestone development. Her height was 60 cm, weight 7 kg and head circumference 35 cm. The second female child was born normal. Written informed consent was taken from the individuals of the family for this study.

### Cytogenetic analysis

About 2 ml of peripheral blood was collected from the family members for the cytogenetic evaluation with their informed written consent. All the samples were subjected to lymphocyte culture according to standard cytogenetic protocols.[5] About 50 Giemsa–Trypsin–Giemsa (GTG)-banded[6] metaphase chromosomes were analyzed for all the individuals of the family. In individuals with abnormalities, a total of 100 metaphases were analyzed.

### Fluorescence *in situ* hybridization (FISH) and microscopy

FISH was performed on metaphase spreads derived from the patient’s lymphocytes. Hybridizations were performed using a chromosome 8-specific DNA probe (WCP 8) Spectrum Green (Vysis, Naperville, IL, USA) and a chromosome 18-specific DNA probe (WCP 18) Spectrum Red according to the Vysis manufacturer’s protocol. Targeted metaphase slides were counterstained with 20 *μ*l of 4-6-diamino-2-phenylinodole (DAPI) (10 g/ml). Bright light and fluorescence microscopy was performed using a Zeiss Axioscope microscope (Zeiss, Jena, Germany). A triple filter set (Vysis) was used for simultaneous detection of DAPI, Spectrum Green and Spectrum Red signals. Several images were analyzed using a Cytovision-automated karyotyping system (Applied Imaging, Santa Barbara, CA, USA). Every slide was scored by a minimum of three cytogeneticists.

## Results

**Figure 1:**
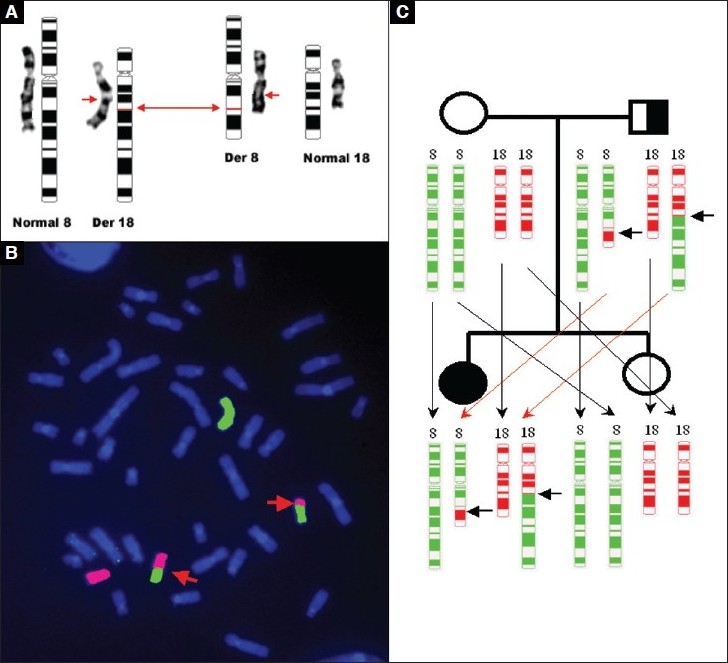
(A) Giemsa-Trypsin-Giemsa banding and ideogram results show autosomal-balanced reciprocal translocation involving chromosomal regions 8q and 18q. The arrows indicate the breakpoints on derivative chromosomes 8 and 18. (B) Fluorescence in situ hybridization of metaphase spread with the Vysis WCP DNA probes, which hybridize chromosome 8 (Spectrum Green) and chromosome 18 (Spectrum Red). The arrows indicate the derivative chromosomes 8 and 18. (C) Pedigree demonstrates characteristics of autosomal-recessive inheritance

Cytogenetic evaluation of the GTG-banded metaphases from a lymphocyte culture showed a balanced reciprocal translocation involving chromosomes 8 and 18 in the proband with a karyotype of 46,XX,t(8;18)(q22.1;q22) [Figure 1A]. To trace the origin of the translocation, cytogenetic analysis from peripheral blood of all the family members was performed, which revealed that the mother and sister of the proband showed a normal female karyotype, whereas the father of the proband was found to be a carrier of the balanced reciprocal translocation with 46,XY,t(8;18)(q22.1;q22) karyotype. Further confirmation of this balanced reciprocal translocation was performed by FISH using WCPs for chromosomes 8 and 18 [Figure 1B].

## Discussion

The sequencing of the entire human genome has revealed the location of an estimated 50,000 genes approximately, although very little is known about their role in human morbidity. Physical mapping of genes involves chromosome abnormalities and variations as two important factors. Hence, to establish genotype–phenotype relationships, a large-scale strategic approach will be of greater importance. Linking up the association of disease consistently with chromosome abnormalities such as deletions, duplications and translocations would be the simple way of mapping disease genes. Mapping of von Recklinghausen neurofibromatosis (NF1) to chromosome 17[7] and the rare congenital disorder campomelic dysplasia (CMPD1), also mapped to chromosome 17[8–11], are good examples of this strategy. Molecular characterization of chromosomal breakpoints in carriers of balanced translocation would be one of the vital strategies to be implemented. Thus, disease-associated chromosome rearrangements[12] that truncate, delete or otherwise inactivate specific genes have been instrumental in the positional cloning of many disease genes.[4] In this paper, we report an interesting case with apparently inherited reciprocal-balanced translocation that has resulted in delayed development of milestones. Delayed milestones or developmental delays are defined as a lag in the child’s development compared to the established standard normal ranges for his or her age. From birth to 6 years of age, 8% of all children show delays in one or more areas of development. The present report indicates that the proband had developmental lags in the first year of her life. She could not crawl by 8 months of age and walk by the middle of the second year; thus, 5 or 6 months behind the normal standard schedule in reaching these milestones, indicating developmental delay regarding mobility. The proband was not speaking words or sentences by her third birthday, while almost all normal children begin to speak their first words before or at the age of 18 months, and by age 3, the majority of children speak short sentences. At toddlerhood, the proband was found to be reserved and less adventuresome as compared to a normal kid who begins to explore the environment with avid curiosity and immense energy to strike out independently and master new skills. Parents had no history of delayed milestones in their childhood.

Balanced chromosomal translocations may cause damage or alteration of the functional genes at the breakpoints of the defective chromosomes resulting in the disease phenotype.[13] It was described previously that children who inherit reciprocal balanced translocation from one of the parents show association with congenital malformation.[14–16] The present case report, however, is the first report of paternal inheritance of balanced reciprocal translocation involving chromosomes 8 and 18 at their respective breakpoint, which shows an association with delayed milestones and which has not been reported previously.

Couples with balanced reciprocal translocations could have a 50% risk of having spontaneous abortions and a 20% risk of having children with unbalanced karyotype.[17] On the basis of the chromosomes involved and on the location of breakpoints, the production of normal, balanced or unbalanced gametes is decided.[18,19] The configuration of the hexavalent at the pachytene stage of meiosis was predominantly used to consider the pattern of segregation; only two configurations result in a normal or balanced gamete karyotype.

In carriers of balanced translocation, the possible reason for the association of congenital malformations could be gene inactivation or disruption at the breakpoint or a position effect.[20] However, in the case of inherited reciprocal translocation seen in the proband, the breakpoint could inactivate genes, subsequently unmasking a recessive allele inherited from the other parent.[21] The other possible reason could be the occurrence of unequal crossing-over during meiosis that may have resulted in submicroscopic duplications or deletions, as proposed by Jacobs.[20] The present case report shows the inheritance of autosomal balanced reciprocal translocation by the proband from her carrier father who is phenotypically normal. This could be explained as an autosomal-recessive inheritance where, unlike the normal child, there is inheritance of the dominant alleles from both the parents and the recessive alleles along with defective alleles inherited to the proband [Figure 1C].

The gene *COH1* that maps at 8q22.2 encodes a potential transmembrane protein that functions in vesicle-mediated transport and sorting of proteins within the cell. This protein plays an important role in the development and the function of the eye, hematological system and central nervous system.[22] Mutations in this gene have been associated with Cohen syndrome.[23] Another important gene *NEDD4L* mapped to 18q21 is the candidate gene for autosomal-dominant orthostatic hypotensive disorder. Also, *NEDD4L* showed linkage evidence for a susceptibility locus for bipolar affective disorder.[24] Hence, disruption of the gene or group of genes at this breakpoint suggests a cause for delayed developmental milestones. The possible reason leading to delayed milestones may be due to the consequence of the abnormality described in the patient. Hence, further analysis of the breakpoints and molecular characterization of these genes might help in understanding the basis of delayed development of milestones. In view of an increased risk of having congenitally abnormal children, carriers of balanced reciprocal translocation should, therefore, be advised to seek genetic counseling. The genetic counseling for a balanced translocation carrier is often difficult and may require some caution, especially when the fetal karyotype is balanced.[16] Bonthron *et al*.[24] raised this warning in their report of *de novo* submicroscopic deletion of an inherited Robertsonian translocation. Hence, carriers of balanced translocation should be counseled for increased risk of birth defects in their offspring due to *de novo* submicroscopic rearrangements, and reproductive management is performed accordingly.
